# Variability in proximate composition, phytochemical traits and antioxidant properties of Iranian agro-ecotypic populations of fenugreek (*Trigonella foenum-graecum* L.)

**DOI:** 10.1038/s41598-023-50699-9

**Published:** 2024-01-02

**Authors:** Ziba Bakhtiar, Mohammadreza Hassandokht, Mohammad Reza Naghavi, Mohammad Hossein Mirjalili

**Affiliations:** 1 Department of Horticultural Sciences, College of Agriculture and Natural Resources, University of Tehran, Karaj, Iran; 2 Division of Biotechnology, Department of Agronomy and Plant Breeding, College of Agriculture and Natural Resources, University of Tehran, Karaj, Iran; 3 Department of Agriculture, Medicinal Plants and Drugs Research Institute, Shahid Beheshti University, Tehran, 1983969411 Iran

**Keywords:** Plant sciences, Chemistry

## Abstract

Fenugreek (*Trigonella foenum-graecum* L.) is a multi-use annual forage legume crop that is widely used in food products such as syrup, bitter run, curries, stew, and flavoring. In the present study, morphological traits, proximate composition (moisture, crude fibre, protein, fat, carbohydrate, and energy value), total phenol and total flavonoid contents, and antioxidant properties of 31 Iranian agro-ecotypic populations of the plant was investigated. Among the leaf and seed samples studied, the seeds exhibited the high ash (3.94 ± 0.12%), fat (7.94 ± 0.78%), crude fibre (10.3 ± 0.25%), protein (35.41 ± 1.86%), and carbohydrate (50.5 ± 1.90%) content. In general, more energy value (kcal/100 g) was also obtained from the seed (318.88 ± 1.78–350.44 ± 1.27) than leaf samples (45.50 ± 1.32–89.28 ± 0.85). Antioxidant activity and power of leaf samples were ranged from 67.95 ± 0.05‒157.52 ± 0.20 μg/ml and from 45.17 ± 0.01‒361.92 ± 0.78 μmol Fe^+2^ per g dry weigh, respectively. Positive linear correlations between antioxidant activity and total phenolic compounds were observed. A significant correlation between proximate composition (dependent variable) and some morphological features (independent variable) was observed. Considerable variability in the studied traits among the plant samples can be interestingly used in further food and production systems.

## Introduction

The Fabaceae (legumes) is the third-largest flowering plant family that comprises 750 genera and approximately twenty-thousand species^1^. The majority species of Fabaceae are herbaceous, though some are trees and shrubs. Fabaceae is distributed across the globe^2^. Beans (*Phaseolus* spp.), garden pea (*Pisum sativum* L.), broad bean (*Vicia faba* L.), green gram (*Vigna radiata* L.), lentil (*Lens culinaris* Medik.), lupins (*Lupinus* spp.), alfalfa (*Medicago sativa* L.), clovers (*Trifolium* spp.), and fenugreek (*Trigonella* spp.) are the most important legumes in the world^3^. The members of Fabaceae play an important role in the human diets, folk medicine, nutritional and pharmaceutical industries, and animal health throughout the globe^4^.

The genus *Trigonella* L., a member of the tribe Trifolieae^5^, includes about 135 species, which *T. corniculata* Sibth. & Sm*.* and *T. foenum-graecum* L. are the major and widespread species^6^. *Trigonella foenum-graecum*, the most interesting and economically important species, is an annual forage legume with considerable genetic diversity across the world. The plant has 20–130 cm high, straight, branching, the pods 10–18 cm long with a slight curvature, that turns straw when ripe, and containing10–20 rectangular to rounded seed with brown or olive green^7^. Many efforts have been conducted to produce a large number of the plant varieties and mutants with high productivity, rapid growth, seed quality, proximate composition, and nutritional values so far^8–10^.

The warm and semiarid area of the Mediterranean Europe has always been a traditional fenugreek producing region^11^. The plant is currently growing across the Indian subcontinent, China, south east and west of Asia, north of Africa, Russia, Australia, western Canada, and Argentina^12,13^. Cultivation of the plant in the low input agricultural regions as a spice, herb, and forage for the pharmaceutical and food industries has also been reported^14^.

Fenugreek, known as *Shanbalileh* in Persian, is a multipurpose crop for its leaves and seeds that are rich in protein (25.5%), carbohydrates (45‒60%), mucilage (20%), amino acid (4-hydroxyisoleucine) (0.09%), vitamins, unsaturated fatty acids such as linoleic acid (37%) and α-linolenic acid (28%), steroidal sapogenins (diosgenin) (1.7%), pyridine alkaloids (trigonelline) (0.38%), flavonoids (quercetin), coumarins, essential oil (0.015%), and minerals (Fe, Cu, and Zn)^15–17^.

Medicinal properties of the plant such as gastric stimulant, anti-diabetic, anti-cancer, hepatoprotective, antioxidant, antibacterial, immunomodulatory, and hypocholesterolemic effects have been reported so far^18–20^. In the North Africa such as Egypt, the plant seeds are added to bread as a supplement of wheat and maize to control blood sugar levels^7^. The plant sprouting seeds and microgreens (cooked and fresh) are rich in vitamin C and antioxidants which are potent in preventing many diseases, whereas microgreens can be easily cultivated and it takes minimal demands on the resources^21^. Fenugreek has a strong, pleasant, and quite peculiar odor reminiscent of maple^14^.Today, due to changing in food habits of people, fenugreek is welcomed because the seed of the plant is one of the important components of many curry preparations and is used to colour and flavour food, and help digestion^7^.

Natural antioxidants such as flavonoid and phenolic compounds that are obtained from fruits, vegetables, oil seeds and herbs play an important role to prevent of many cancers, and have been widely used in food and pharmaceutical industries^13,22^. So, looking for a new source of antioxidants among natural or cultivated plant populations can be interesting for the researchers throughout the world.

Fenugreek is becoming increasingly popular and enters the market labeled as nutritious, functional and healthy food^15^. Due to increasing demand for healthy and functional food, the present study was aimed to explore variations in morphological characteristics, proximate composition, total flavonoid content (TFC), total phenol content (TPC), and radical scavenging properties of 31 Iranian agro-ecotypic populations (AEPs) of the plant using a range of multivariate statistical methods. These findings can be considered for further commercial exploitation of fenugreek in food industries. The plant materials can be also interesting for the producers to develop new food with high nutritional and antioxidant potential.

## Results and discussion

### Morphological features

The present data indicated a wide variability among the studies AEPs (Table 1). Comparison of quantitative and qualitative parameters for each ecotype showed significant (p < 0.05) differences correlated principally to origin and ecological conditions. Number of twin pods had the highest coefficient of variation (CV = 14.32%). Petropoulos^7^ noted that twin pods in fenugreek indicative of high diosgenin content in the seed. Plant weight ranged between 8.30 ± 0.7 and 73.50 ± 2.4 g with an average of 34.54 g. Leaf color was light-green (35.48%), green (32.26%), and dark-green (32.26%) (Table 2). The duration from the seed sowing to harvest time was 70.20 ± 2.1 to 120.00 ± 0.5 day in all studies AEPs. Mean values for the number of seed per pod and pod number in plant were 10.76 and 21.41, respectively. Also, twin pods were observed in the AEPs with origin of center of Iran (0.6 ± 0.2–12.44 ± 0.1). The weight of 1000 seed ranged between 5.64 ± 0.0 and 20.27 ± 0.1 g with an average of 12.53 g. Seed shape was round (6.45%), elliptic (25.81%), ovate (22.58%), cubic (35.48%), and rectangular (9.68%), while seed color was light-brown (65.29%), brown (22.58%), and olive (16.13%). Seed surface was wrinkled (64.52%) and the other was smooth (35.48%). Approximately, the seed color was light-brown and the seed surface was wrinkled in most of the AEPs. Panwar et al.^23^ reported high significant differences between Indian *T. foenum-graecum* varieties for all the studied characteristics such as pods per plant, plant height, and seed weight per plant and high significant positive correlation of plant height and pod number per plant on seed yield. Mean values of Indian *T. foenum-graecum* varieties for 80 percent maturity, seed weight per plant, and plant height were 128.49%, 13.09 g, and 57.50 cm, respectively. Yimam et al.^24^ also recorded the significant (p < 0.05) difference among *T. foenum-graecum* genotypes for most of the agro-morphological traits examined in Ethiopia as the second most diverse country. The quantitative and qualitative traits of the studies AEPs of fenugreek are in agreement with previous investigations from other countries^23,25–29^. In a study, the seed number per pod, pod number per plant, and 1000 seed weight were measured as 13.6, 5.7, and 7.3 g for the samples collected from Miandoab, Urmia province in Iran, respectively^30^. The studied AEPs had significant phenotypic variation and among them that some high-yield ecotypes in seeds and aerial parts can be planted and then used in food industry.

**Table 1:** Phenotypic variability among the 31 agro-ecotypic populations of *Trigonella foenum-graecum*.

No	Morphological traits	Unit	Min	Max	Mean	SD	CV (%)^a^
1	Plant weight	g	8.30 ± 0.7	73.55 ± 2.4	34.54	19.67	1.45
2	Leaf color	Code	1.00	3.00	1.97	0.82	5.08
3	Sowing to harvest time	Date	70.20 ± 2.1	120.00 ± 0.5	95.40	19.48	3.13
4	Seed number per pod	Number	4.91 ± 0.6	18.12 ± 1.0	10.76	3.64	9.30
5	Pod number per plant	Number	6.9 ± 1.0	64.69 ± 1.6	21.41	13.65	11.68
6	Number of twin pods	Number	0.00 ± 0.0	12.44 ± 0.1	1.40	2.33	14.32
7	1000 seed weight	g	5.64 ± 0.0	20.27 ± 0.1	12.53	4.57	2.98
8	Seed shape	Code	1.00	5.00	3.15	1.11	3.17
9	Seed color	Code	1.00	3.00	1.57	0.76	6.35
10	Seed surface	Code	1.00	2.00	1.36	0.48	0.37

Note: Data are mean ± SD (standard deviation).

^a^CV, coefficient of variation.

**Table 2:** Frequency distribution for the measured qualitative morphological traits in 31 agro-ecotypic populations of *Trigonella foenum-graecum.*

Morphological trait	Frequency (%)				
	1	2	3	4	5
Leaf color	Light-green (35.48)	Green (32.26)	Dark-green (32.26)	‒	‒
Seed shape	Round (6.45)	Elliptic (25.81)	Ovate (22.58)	Cubic (35.48)	Rectangular (9.68)
Seed color	Light-brown (65.29)	Brown (22.58)	Olive (16.13)	‒	‒
Seed surface	Wrinkled (64.52)	Smooth (35.48)	‒	‒	‒

### Proximate composition

**Figure 1:**
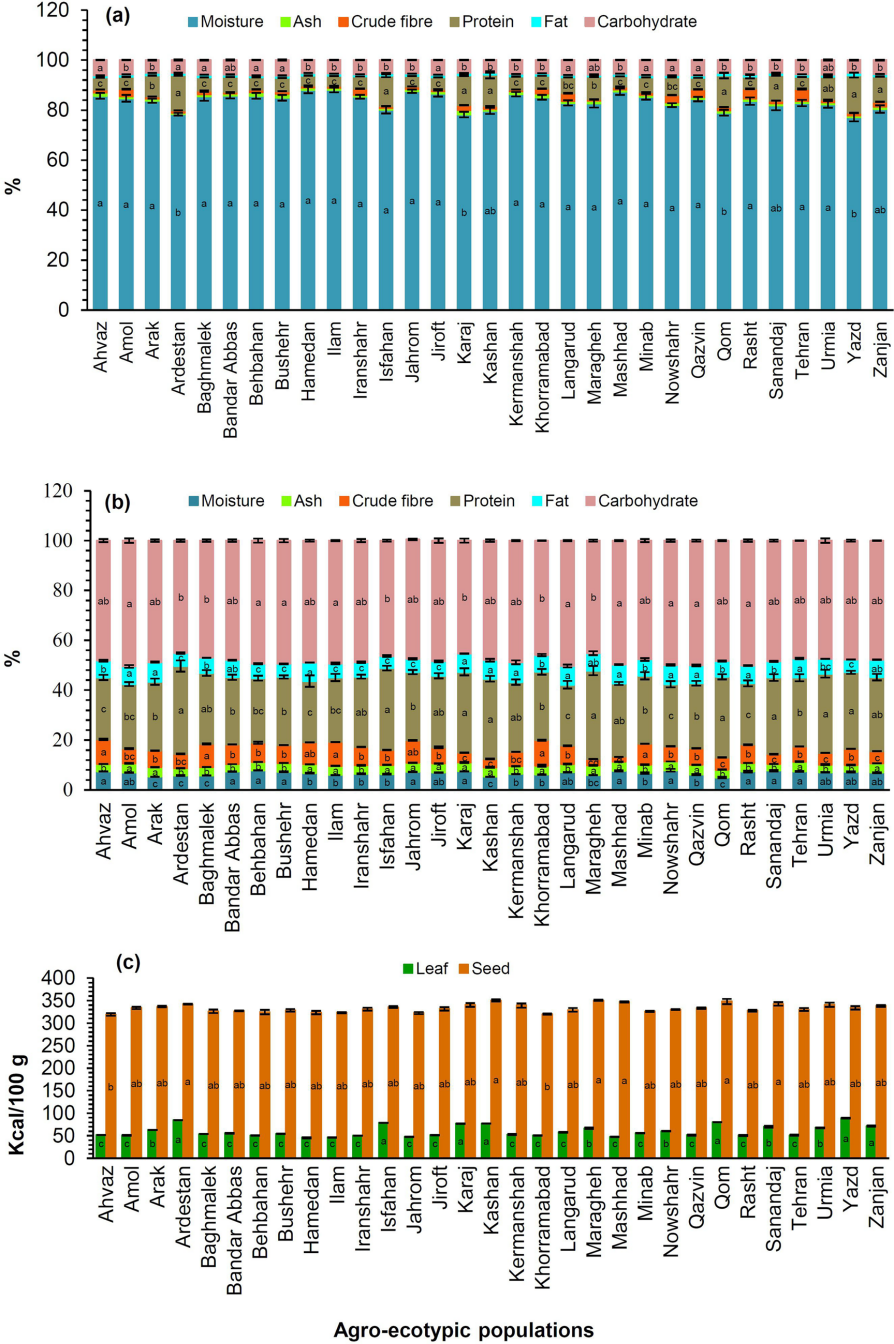
Histograms of proximate composition for leaf (**a**), seed (**b**), and energy value of leaf and seed (**c**) for 31 agro-ecotypic populations of *Trigonella foenum-graecum*.

The proximate composition of the leaf and seed of all AEPs studied including content of moisture, crude fibre, protein, fat, and carbohydrate as well as energy value were measured (Fig. 1a–c). Significant (p < 0.05) and non-significant differences in proximate composition of both materials among plant samples studied were observed. The moisture content of the leaf was ranged from 77.11 ± 1.62% in Yazd ecotype to 88.10 ± 1.27% in Hamedan ecotype, while the lowest and highest moisture content in the seed samples were determined in Qom (4.95 ± 0.28%) and Nowshahr (7.87 ± 0.55%) ecotypes, respectively. The highest amount of ash was found from the leaf of Karaj ecotype (1.72 ± 0.05%) and the seed of Qazvin ecotype (3.94 ± 0.12%), respectively. A significant (p < 0.05) difference in crude fibre content was observed among the seed samples (1.97 ± 0.01–10.3 ± 0.25%). The fat content of leaf samples varied from 0.77 ± 0.05 to 1.06 ± 0.03%, while this content for seed samples ranged from 4.81 ± 0.34 to 7.94 ± 0.78%. Fat content of the plant leaf (1.00%) and seed (6.3%) has been previously reported^31,32^. The leaf of Bushehr ecotype (6.15 ± 0.25%) and the seed of Amol ecotype (50.5 ± 1.90%) had the highest content of carbohydrate. The moisture, crude fibre, ash, fat, and carbohydrate content of the plant seed were at the similar level as shown by El Nasri and El Tinay^33^.

The maximum protein content of the leaf samples was found in Yazd ecotype (15.00 ± 0.94%) followed by Ardestan (14.16 ± 0.22%), Qom (12.63 ± 1.17%), Kashan (12.50 ± 1.43%), and Isfahan (12.13 ± 0.55%) ecotypes, while in the seed samples, Maragheh ecotype (35.41 ± 1.86) followed by Ardestan (35.21 ± 2.29%), Isfahan (32.76 ± 0.94%), Karaj (32.34 ± 1.44%), Qom (32.30 ± 1.03%), and Kashan (32.12 ± 1.12%) ecotypes had the highest protein content. In the plant leaf, Gupta et al.^34^ observed slight higher content of protein (15.60%) while, in the contrast, Srinivasan^35^ reported relative lower content of protein (4.4%). A wide range of protein content (21.28‒28.00%) has also been reported in the plant seeds^35,36^. The results revealed that Iranian AEPs of fenugreek can be considered as a potential source of protein for further use in functional food and products. Differences in proximate composition of fenugreek leaf and seed from different populations and origins have already been reported^32,37–40^. Audu et al.^41^, also found the seeds of *T. foenum-graecum* from Nigeria contain 3.54 ± 0.42% moisture, 3.37 ± 0.08% ash, 19.3 ± 0.35% proteins, 4.14 ± 0.11% fat, 7.05 ± 2.24% crude fibre.

The highest and lowest energy value (kcal/100 g) of the plant leaf were determined in Yazd (89.28 ± 0.85) and Hamedan (45.50 ± 1.32) ecotypes, respectively. This trait for the seed samples was at the highest value in Maragheh ecotype (350.44 ± 1.27 kcal/100 g). Energy value is calculated when fat, protein, and carbohydrate values are available for a food, so this value mainly depends on the fat content^42^. Energy value of raw and mature seeds of the other Fabaceae members such as peanut, soybean, black bean, green bean, and alfalfa has been reported as 567, 446, 341, 31, and 23 kcal/100 g based on DW, respectively (USDA 2019).

Pandey and Awasthi^43^ obtained better results when they changed processing methods of the plant seed. They showed that the plant raw seeds contain 6.30% moisture, 32.70% protein, 6.00% crude fibre, 4.80% fat, 3.70% ash, and 46.1% carbohydrate, while the protein content increased from 32.7 to 41.2% and 36.8% after germination and roasting, respectively. Increased protein content of the plant germinated seeds can be related to the reduction of seed nitrates into protein or ammonium compound, and enzymatic synthesis of protein as well, which is in agreement with other earlier reports^44,45^. According to these findings, making beverages from roasted fenugreek seeds as a coffee substitute and also sprouted seeds as microgreens which has become popular nowadays, is more useful and energizing for high protein diet.

### Total phenol and total flavonoid contents and radical scavenging capacity

**Figure 2:**
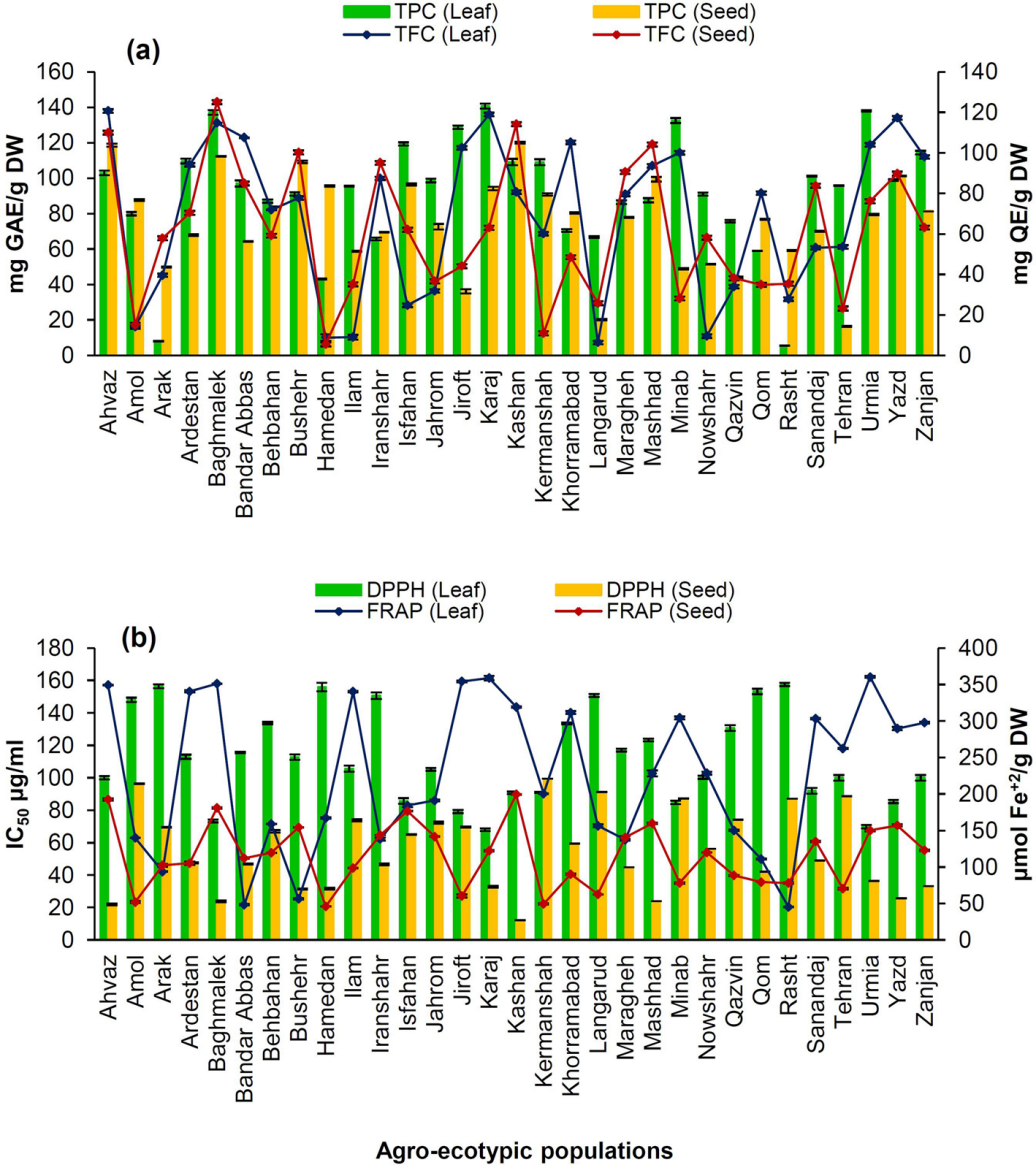
Histograms of total phenol content (TPC), total flavonoid content (TFC) (**a**), and radical scavenging activities (**b**) of leaf and seed extracts from 31 agro-ecotypic populations of *Trigonella foenum-graecum*.

The TPC, TFC, and antioxidant properties of the plant samples are presented in Fig. 2. Nowadays, looking for natural sources of antioxidants has noticeably increased^46^. In the current study, the highest FRAP (361.92 ± 0.78 μmol Fe^+2^/g DW) and DPPH (67.95 ± 0.03 IC_50_ μg/ml) values as well as the highest TPC (140.76 ± 1.43 mg GAE/g DW) from the plant leaf samples were observed in Karaj ecotype. Uras Güngör et al.^12^, have been obtained the same results in the aerial parts *T. velutina* Boiss. According to the pervious study^47^, TPC were highly correlated with DPPH and ferric reducing antioxidant power values. However, Gupta and Prakash^48^ found that the TPC and antioxidant activity (IC_50_) of fenugreek leaf extract were 158.3 mg tannic acid equivalent/100 g and 27.69 mg/ml, respectively.

The seed samples extract showed higher antioxidant activity (IC_50_) ranged from 12.21 ± 0.09 to 99.54 ± 0.01 μg/ml. The same results have been reported in *T. arabica* Delile and *T. berythea* Boiss. & C.I.Blanche^49^. The same ranges of antioxidant activity for different varieties of *T. foenum-graecum* have also been reported^50^. The highest TPC (mg GAE/g DW) of seed extracts was observed in Kashan ecotype (120.04 ± 0.58) followed by Ahvaz ecotype (118.69 ± 0.84), which was similar results obtained for *T. cylindracea* Desv.^12^. Total phenol content of 85.88 mg GAE/g and antioxidant activity of 60% have been reported from the seed extract of fenugreek^18^. In accordance with the report of Lohvina et al.^51^, the TPC and IC_50_ value of fenugreek seed extract were 120.30 mg GAE/g DW and 100.00 μg/ml, respectively. Mnafgui et al.^52^ recently evealed that total phenol and total flavonoid values in fenugreek shoots from Iran as 11 mg GAE/g DW and 1.5 mg QE/g DW, respectively.

The highest TFC of leaf and seed extracts was determined in Ahvaz (120.70 ± 0.88 mg QE/g DW) and Baghmalek (125.12 ± 0.09 mg QE/g DW) ecotypes, respectively. The leaf extracts of eighteen and seed extracts of twelve AEPs had 2-fold or more TFC than the other AEPs. The leaf extract of Rasht ecotype exhibited the lesser TPC (5.49 ± 0.06 mg GAE/g DW), and had the least antioxidant properties (IC_50_ = 157.52 ± 0.20 IC μg/ml and FRAP 45.17 ± 0.01 μmol Fe^+2^/g DW) compared to the other AEPs. A wide variation of TPC, TFC and antioxidant activity of the plant leaf extracts have already been reported^53–55^. Baba et al.^56^ have also found the leaf extract of *T. foenum-graecum* is the better source of antioxidant compounds than seed.

### Principal component analysis and correlations between the variables

**Figure 3:**
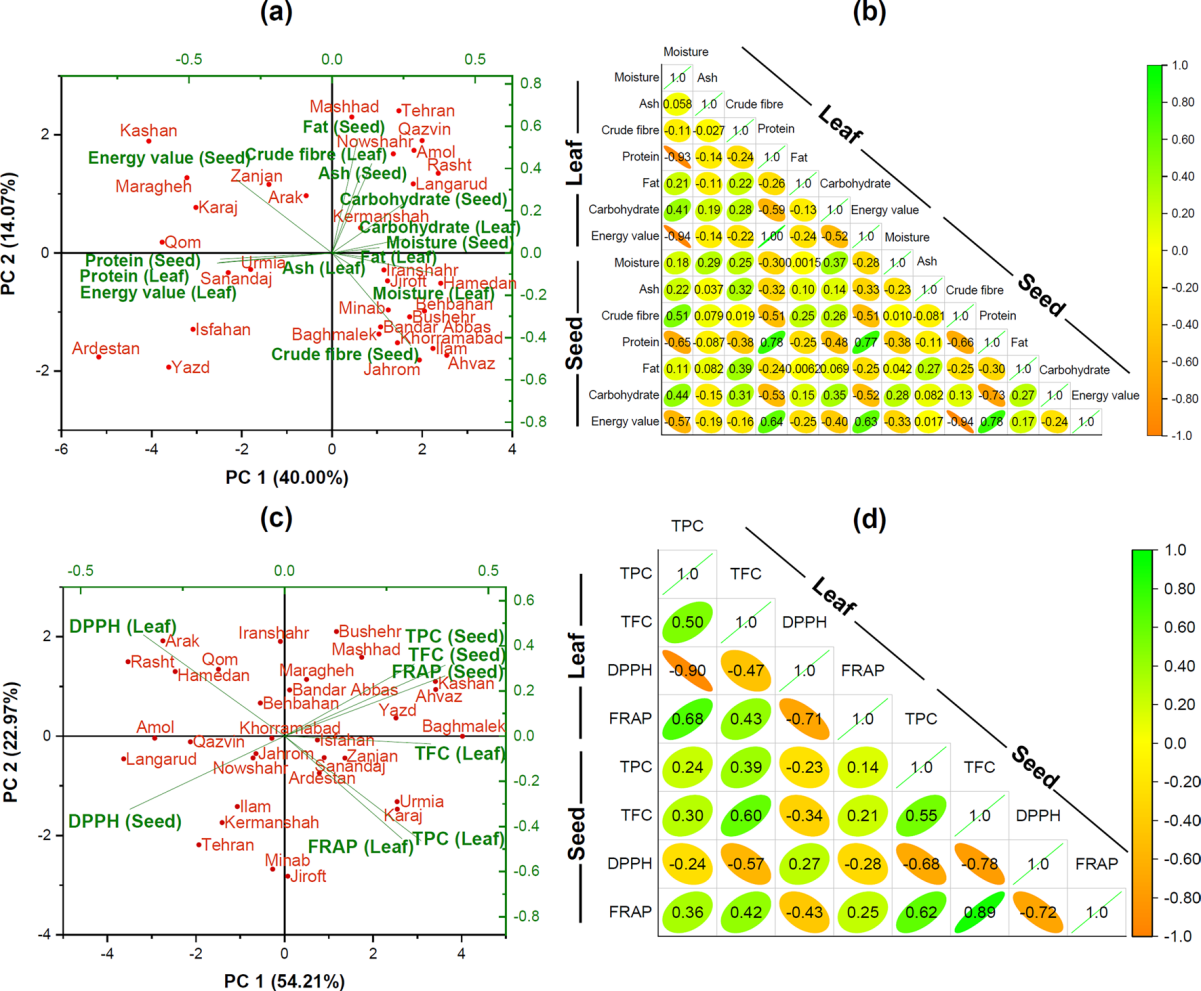
Bi-plot graph and correlation plot for the first and second principal components based on the proximate composition (**a**, **b**), and total phenolic, flavonoid content and antioxidant activities (**c**, **d**) for 31 agro-ecotypic populations of *Trigonella foenum-graecum* (Significant level: 0.05).

Principal component analysis (PCA) was used to establish the association between proximate composition of leaf and seed of the studied AEPs. The first and second PCA for proximate composition yielded 40.00% and 14.07% of the total variance, respectively (Fig. 3a). Consistent with the bi-plot graph, four groups of AEPs were identified using the proximate composition for leaf and seed. Along axis 1 of the graph, eight ecotypes (Amol, Kermanshah, Langarud, Mashhad, Nowshahr, Qazvin, Rasht, and Tehran) were grouped on the positive region and strongly contributed to crude fibre and carbohydrate of leaf and moisture, ash, fat, and carbohydrate of seed. Six other ecotypes (Arak, Karaj, Kashan, Maragheh, Qom, and Zanjan) were negatively correlated with energy value of seed. Along axis 2 of the graph, twelve further ecotypes (Ahvaz, Baghmalek, Bandar Abbas, Behbahan, Bushehr, Hamedan, Ilam, Iranshahr, Jahrom, Jiroft, Khorramabad, and Minab) formed a separate group on the positive region of the PC2 axis and were associated with moisture, ash, and fat of leaf and crude fibre of the seed. The remaining ecotypes (Ardestan, Isfahan, Sanandaj, Urmia, and Yazd) formed an extra group in the negative section of PC2 axis and were related to protein and energy value of leaf and protein of seed. Along negative section of PC2 axis, Ardestan, Isfahan, and Yazd ecotypes were the farthest samples from the center. The highest protein contents of leaf and seed were found in the ecotypes.

The results of the correlation analysis between the proximate composition of leaf and seed were shown in Fig. 3b. Positive and negative correlations were observed between proximate composition of leaf and seed. The correlation matrix showed the relationships among the leaf protein with seed protein (*r* = 0.78) and seed energy value (*r* = 0.64) and also, negative correlation with crude fibre (*r* = − 0.51) and carbohydrate (*r* = − 0.53) of seed. A positive correlation energy value of leaf was recognized between the amount of protein (*r* = 0.77) and energy value (*r* = 0.63), and it had an intermediate correlation with crude fibre (*r* = − 0.51) and carbohydrate (*r* = − 0.52) of seed. The correlation of leaf moisture with protein (*r* = − 0.65) and energy value (*r* = − 0.57) was found to be moderately high and negative, while it was positive (r = 0.51) with crude fibre of leaf. Significant negative correlations between protein and carbohydrate (*r* = − 0.59), as well as carbohydrate and energy value (*r* = − 0.52) of leaf were observed, which indicated that fenugreek ecotypes with high protein and energy value may likely to be low in carbohydrate. The leaf protein had a strong positive with leaf energy value (*r* = 1.00). The ash and fat of leaf and seed had no significant relations with other parameters. The “*r*” value for moisture and protein (*r* = − 0.93) and leaf energy value (*r* = − 0.94) was negative and high, indicating a notable association among these three compounds in leaf of the studied AEPs. Positive correlation of protein with energy value (*r* = 0.78) and also negative relation with carbohydrate (*r* = − 0.73) of seed was observed. Crude fibre of seed showed high negative and significant correlation with protein (*r* = − 0.66) and energy value (*r* = − 0.94). High negative correlation between crude fibre content and energy value of the seed revealed that the AEPs studied with low level of crude fibre would also provide more energy for body.

Correlation analysis exhibited that, the proximate composition of leaf had a significant positive and negative association with some measured parameters of seed. Leaf growth as a source organ can be directly affected the proximate composition of the seed. Also, different growth conditions can be changed the metabolic pathways which led to an imbalance in vegetative and reproductive growth^57^, so the synthesis and accumulation of protein, carbohydrate, and fat in the plant organs will be limited, although the mechanisms require further study. In some case, there was a slight difference between the results obtained and earlier reports which can be probably due to different geographical origins, genotype, cultivated regions, environmental parameters (temperature, pressure, humidity, and wind), maturity stage, storage conditions, and extraction methods^32^.

According to Fig. 3c, the first and second PCA for TPC, TFC, and antioxidant activity scored 54.21% and 22.97% of the total variance, respectively. Along axis 1, Ahvaz, Bandar Abbas, Bushehr, Kashan, Maragheh, Mashhad, and Yazd ecotypes formed a group on the positive section and were characterized by TPC, TFC, FRAP of seed extract. Along axis 2, eight AEPs (Ardestan, Baghmalek, Isfahan, Jiroft, Karaj, Sanandaj, Urmia, and Zanjan) were related with higher antioxidant capacity (FRAP), TPC, and TFC of leaf extract. None of the measured parameters contributed to the sixteen other AEPs, which formed another group in the negative section of axis II (PC2). The leaf and seed extract of these AEPs had high IC_50_ indicating low antioxidant activities.

Pearson’s correlation analysis exhibited a significant relation between antioxidant activity, TPC, and TFC (Fig. 3d). Moderately positive and negative correlations were observed between leaf and seed extract. There was correlation between TFC of leaf extract and TFC (*r* = 0.60) of seed extract, and negative correlation between TFC of leaf extract and DPPH (*r* = − 0.57) of seed extract. The TPC values of the leaf extract were moderate to high correlated with TFC and FRAP with R values of 0.50 and 0.68, respectively, and also strong negative correlation with DPPH of (*r* = − 0.90). The TPC of seed extract had the highest positive correlations with the following cases as TFC (*r* = 0.55) and FRAP (*r* = 0.62), and negative correlation with DPPH (*r* = − 0.68) of seed extract. Similarly, high significant correlation was revealed between TFC and FRAP (*r* = 0.89) in the seed extract, while this value had negative and significant correlations with DPPH (*r* = − 0.78). Our results revealed that the phenolic and flavonoid components were remarkably contributed to the antioxidant property of fenugreek. The other researchers have also declared a positive and significant association between antioxidant activity and the plant total phenol and total flavonoid conten^54,58, 59^. Himalian and Singh^60^ reported that flavonoids increase the antioxidant activity of the plant.

A significant negative correlation between DPPH and FRAP in the leaf extract (*r* = − 0.71) and seed extract (*r* = − 0.72) confirms the authenticity of the antioxidant potential of different fenugreek AEPs. Moreover, antioxidant capacity of various extracts may not only be related to TPC and TFC, but also the presence of the other biochemical and phytochemical components has been considered by many researchers^61,62^.

It has also been claimed that genetic factors, climatic conditions, harvest time, and post-harvest processes can be affected the TPC, TFC, and antioxidant activity of plant samples as a result of changing metabolic pathway^46,63^.

The proximate composition data was grouped the ecotypes originated from the north of Iran in one group characterized by high amounts of carbohydrates, ash and fat. The ecotypes that originated from the south of Iran were also placed in one group, which were characterized by the high content of leaf moisture and crude fiber in the seeds, while the ecotypes from the center and northwest of Iran were clustered in another group based on their high content of energy and protein in the leaves and seeds. Based on higher TPC, TFC, and antioxidant activity, the plant ecotypes originated from the center and south of Iran were separated from the northern ecotypes with lower values. Considering that the cultivation conditions of the studied ecotypes were similar, the variation in the observed proximate and phytochemical compositions could depend on intrinsic factors.

### Multiple regression data

Proximate composition as dependent variable, demonstrated a significant correlation with several morphological variables as independent variable (Table 3). No direct correlation between TPC, TFC, DPPH, and FRAP with morphological characters in the studies plant samples was found. Plant weight and seed surface were found to be correlated with more than one parameter of proximate composition.

**Table 3:** Proximate composition associated with morphological characters using multiple regression analysis.

Proximate composition	Morphological parameter	*r*	*R*^2^	Standardized beta coefficients	*t* value	*p* value
Moisture (Leaf)	Plant weight	0.754^a^	0.569	‒ 0.738	‒ 6.380	0.000
	Seed color	0.792^b^	0.627	‒ 0.241	‒ 2.081	0.047
Crude fibre (Leaf)	Seed surface	0.423^a^	0.179	0.423	2.515	0.018
Protein (Leaf)	Plant weight	0.727^a^	0.529	0.727	5.702	0.000
Fat (Leaf)	Seed number per pod	0.397^a^	0.158	0.397	2.330	0.027
Carbohydrate (Leaf)	Plant weight	0.606^a^	0.367	‒0.606	‒ 4.102	0.000
Energy value (Leaf)	Plant weight	0.709^a^	0.502	0.709	5.408	0.000
Moisture (Seed)	Leaf color	0.362^a^	0.131	‒ 0.362	‒ 2.090	0.046
Crude fibre (Seed)	Number of twin pods	0.518^a^	0.268	‒ 0.518	‒ 3.257	0.003
Protein (Seed)	Plant weight	0.517^a^	0.268	0.517	3.257	0.003
Fat (Seed)	Seed surface	0.407^a^	0.166	0.407	2.401	0.023
Energy value (Seed)	Plant weight	0.477^a^	0.227	0.477	2.922	0.007

For instance, plant weight had a positive correlation (p < 0.01) with protein and energy value of leaf (*β* = 0.727 and 0.709) and seed (*β* = 0.517 and 0.477), while had a negative correlation (p < 0.01) with moisture (*β* = − 0.738) and carbohydrate (*β* = − 0.606) of leaf. There was a negative significant correlation between seed color and moisture of leaf (*β* = − 0.241, p < 0.05) and also leaf color with moisture of seed (*β* = − 0.362, p < 0.05). Seed surface was found to be associated with crude fibre of leaf (*β* = 0.423, p < 0.05) and fat of seed (*β* = 0.407, p < 0.05). Besides, seed number per pod was associated with fat of leaf (*β* = 0.397, p < 0.05). Ultimately, number of twin pods showed negative association (p < 0.01) with crude fibre of seed (*β* = − 0.518). This finding is consistent with those reported by Hintz, and Albrecht^64^ and Yassein et al.^65^ who reported that morphological characteristics correlated with seed oil content of Safflower (*Carthamus tinctorius* L.), and crude fibre and protein content of alfalfa (*Medicago sativa* L.) forage. Thus, multiple regression analysis can improve the accuracy of predicting leaf and seed proximate composition such as fat, protein and crude fibre. The use of easily determined parameters such as morphological features may provide the best compromise between prediction accuracy and ease of use. Thus, finding correlations between morphological traits and proximate compositions can be also helped plant breeders to select adequate populations for further herbage and seed quality improvement such as protein, fat, and fibre content.

## Conclusions

Considerable variations in morphological traits, proximate composition, TPC, TFC, and antioxidant activity were observed among the studied *T. foenum-graecum* ecotypes. So, phenotypic traits (plant weight, seed number per pod, pod number per plant, and 1000 seed weight), proximate composition (protein, crude fibre, and fat), phenolic compounds, and antioxidant activity can be considered as index features for further uses in the plant breeding and cultivation programs. The seeds of the studied Iranian fenugreek ecotypes were also contained a good level of protein, fat, and carbohydrate. Also, the TPC and TFC in the leaves and seeds of the AEPs were significantly contributed to their antioxidant capacity. Our findings revealed that the leaf of Karaj ecotype and the seeds of Mashhad ecotype can be interestingly selected for further commercial exploitation in the food industries.

## Materials and methods

### Chemicals

Aluminium chloride, sodium nitrite, sodium hydroxide, sodium carbonate, copper sulfate, potassium sulfate, sulfuric acid, DPPH (1,1-Diphenyl-2-picryl hydrazyl), 2,4,6-tri(2-pirydyl-s-triazine (TPTZ), quercetin, gallic acid (GA), tert-butyl-4-hydroxy toluene (BHT), Folin-Ciocalteu’s reagent, methanol, and* n*-hexane were purchased from Sigma-Aldrich Co. (Buchs, Switzerland).

### Plant materials and cultivation site

The seeds of 31 AEPs of fenugreek collected from twenty-four provinces across Iran were sown in the research farm of Horticultural Research Station, University of Tehran, Mohammadshar, Karaj, Iran (N35° 46′, E50° 55′ at an altitude of 1320 m) in a randomized complete block design (RCBD) with three replications from May to September 2020. An inter- and intra-row distance of 30 cm and 1 m was considered for the planting. The agricultural care and management practices were followed for raising a good field crop including weed control and fertilization are based on scientific advices and recommendations. During the growth period, irrigation was done once a week. The experimental site had a semi-arid climate with 37% average relative humidity. Climate and also soil characteristics of the cultivation site are provided in Table 4. The aerial parts and seeds of AEPs samples were collected on August and September 2020, respectively. The collected samples were shade dried, and voucher specimens have been deposited for all studied AEPs in Herbarium of College of Agriculture and Natural Resources (Herbarium Instituti Agronomici Keredjensis) (HIAK), University of Tehran, Karaj, Iran. A brief description of the AEPs is presented in Table 5.

**Table 4:** Climate and soil physicochemical characters of the cultivation site.

Climate parameters		
Months	MAT (˚C)	MTP (mm)
May	17.6	2.4
June	24.3	0
July	27.5	0
August	27.1	0
September	23.9	0

Soil properties		
Textural	Loamy	
Power of hydrogen (1:10)	7.6	
Electrical conductivity (ds/m)	0.73	
Lime (%)	5.50	
Organic matter (%)	0.80	
Usable Phosphor (mg/kg)	22.3	
Usable Potassium (mg/kg)	350	

MAT, monthly average temperature.

MTP, monthly total precipitation.

**Table 5:** Geographic location of the studied agro-ecotypic populations of *Trigonella foenum-graecum* from Iran.

No	Population name	Voucher number	Geographical parameters		
			Latitude (N)	Longitude (E)	Altitude (m)
1	Ahvaz	HIAK‒6492	31° 20′	48° 40′	10
2	Amol	HIAK‒6493	36° 23′	52° 20′	76
3	Arak	HIAK‒6494	34° 00′	49° 40′	1708
4	Ardestan	HIAK‒6495	33° 20′	52° 25′	1207
5	Baghmalek	HIAK‒6496	31° 31′	49° 51′	917
6	Bandar Abbas	HIAK‒6500	27° 15′	56° 15′	9
7	Behbahan	HIAK‒6497	30° 30′	50° 15′	325
8	Bushehr	HIAK‒6498	28° 55′	50° 55′	4
9	Hamedan	HIAK‒6499	34° 52′	48° 32′	1803
10	Ilam	HIAK‒6501	33° 36′	46° 36′	1427
11	Iranshahr	HIAK‒6502	27° 15′	60° 40′	591
12	Isfahan	HIAK‒6503	32° 39′	51° 43′	1571
13	Jahrom	HIAK‒6504	28° 30′	53° 31′	1050
14	Jiroft	HIAK‒6505	36° 36′	47° 26′	720
15	Karaj	HIAK‒6506	35° 48′	51° 00′	1380
16	Kashan	HIAK‒6507	34° 05′	51° 30′	982
17	Kermanshah	HIAK‒6508	34° 23′	47° 00′	1374
18	Khorramabad	HIAK‒6509	33° 30′	48° 57′	1347
19	Langarud	HIAK‒6510	37° 19′	50° 16′	− 14
20	Maragheh	HIAK‒6511	37° 30′	46° 12′	1477
21	Mashhad	HIAK‒6512	36° 20′	59° 35′	1065
22	Minab	HIAK‒6513	27° 15′	57° 07′	16
23	Nowshahr	HIAK‒6514	36° 40′	51° 30′	− 21
24	Qazvin	HIAK‒6515	36° 15′	50° 00′	1297
25	Qom	HIAK‒6516	34° 40′	51° 00′	932
26	Rasht	HIAK‒6517	37° 20′	49° 58′	8
27	Sanandaj	HIAK‒6518	35° 32′	46° 99′	1463
28	Tehran	HIAK‒6519	35° 44′	51° 30′	1368
29	Urmia	HIAK‒6520	37° 55′	45° 08′	1363
30	Yazd	HIAK‒6521	32° 00′	55° 00′	1230
31	Zanjan	HIAK‒6522	37° 80′	47° 47′	1638

### Declaration statement

We declare that the collection of plant material is in accordance with relevant institutional, national, and international guidelines and legislation.

### Morphological evaluation

The range of phenotypic variability among the AEPs selected was evaluated using 6 quantitative and 4 qualitative characteristics. The data were recorded from ten randomly selected plants of each ecotype for various quantitative traits including number of days from sowing to harvest time, number of seed per pod, number of pod, and twin pods per plant, herb weight (g), and 1000 seed weight (g). The weight of herb and seed was measured using a digital balance with 0.01 g precision (GM152, Sartorius, Germany). Qualitative characteristics including seed and leaf color, seed shape and surface were evaluated based on coding and scoring as presented in Table 2.

### Proximate composition analysis

The proximate composition of leaf and seed of the plant AEPs were analyzed by the Association of the Official Analytical Chemists (AOAC) methods^66^. To determine moisture content, 5 g of herb and seed were dried in oven (UNE600, Memmert, Schwabach Germany) at 103 °C for 24 h. The weight of oven-dried samples was then recorded by digital balance with 0.001 g precision (Quintix 513-1S, Sartorius, Germany). Ash content was obtained by residue samples in a muffle oven (KSL-500X-71, MTI corporation, USA) at 550 °C for 24 h (AOAC method 942.05). Fat extraction was based on the continuous process using a Soxhlet apparatus (SOX406, HANON Systems, South Korea) with *n*-hexane for 4 h at 80 °C (AOAC method 920.39). The protein content was also estimated by Kjeldahl (K1100, HANON Systems, South Korea) method (AOAC method 954.01). In brief, 100 mg of copper sulfate (CuSO_4_), 500 mg of potassium sulfate (K_2_SO_4_), and 7 ml of concentrated sulfuric acid (H_2_SO_4_) were added to 1 g of each sample. Digestion was carried out for 1 h at 400 °C. After addition of 20 ml solution of NaOH (15 mol/dm^3^), ammonia (NH_3_) was collected into the boric acid (H_3_BO_3_) solution containing the methyl red. Borate anion was titrated with standardized H_2_SO_4_ (0.1 M). Protein values were subsequently obtained by using a factor of 6.25. To determine crud fibre, the sample digestion was carried out by boiling acid and alkali according to the AOAC method (962.09). The total carbohydrate was also obtained by difference.

The energy value was evaluated using the following equation^42^:

$${\text{Energy value }}\left( {{\text{kcal}}/{1}00{\text{ g}}} \right)\, = \,(\% {\text{ carbohydrate\,\,content}}\, \times \,{4})\, + \,(\% {\text{ protein\,content}}\, \times \,{4})\, + \,(\% {\text{ fat\,\,content}}\, \times \,{9})$$

### Extraction and total phenol and total flavonoid contents determination

The dried powdered samples (100 mg) were mixed with methanol (10 ml) and extracted by ultrasonic (E-100-H, Elma, Germany) at 25 °C for 20 min. The extracts were centrifuged (Rotanta 460r, Hettich, Germany) at 15,000 g for 20 min. The supernatants were dried in a rotary evaporator (Heidolph Instruments GmbH, Schwabach Germany) at 35 °C and dissolved in methanol with a final concentration of 1 ml for determination of TPC and TFC.

The method of Folin-Ciocalteu was used to measure TPC^67^. Initially, 100 µl extract was mixed with 200 µl Folin-Ciocalteu solution, and 700 µl sodium carbonate (700 mM). The extract was then placed on a shaker (Rotamax 120, Heidolph, Germany) at 80 rpm for 30 min in the dark condition. The absorbance was read at 765 nm using a spectrophotometer (Bio-Tek Instruments, Inc., USA). Linearity range of the calibration curve was 6.25–1000 μg/ml (*y* = 0.0034x + 0.1955, *R*^2^ = 0.9929). The results were expressed as mg gallic acid equivalent per g of dry weigh (mg GAE/g DW) according to Kamtekar et al.^68^.

For determination of TFC, 25 μl sample solution, 100 μl of distillated water, 6 μl (0.5 M) sodium nitrite, 6 μl aluminium chloride hexahydrate (0.3 M), and 80 μl sodium hydroxide (1.0 M) were pipetted to plate respectively. The solution was incubated in the dark at 25 ± 2 °C for 15 min.

Absorbance was measured at 510 nm against methanol as a blank. Linearity range of the calibration curve was 12.5–1000 μg/ml (*y* = 0.0003x + 0.0442, *R*^2^ = 0.9997). Data were expressed as mg quercetin equivalent QE/g DW (mg QE/g DW) as described previously^68^. Each sample was analyzed in triplicate.

### Antioxidant properties

Antioxidant activity of the plant samples extracts was determined following the method of Akhlaghi and Najafpour-Darzi^69^ with few modifications. For instance, 4.0 ml of 0.1 mM DPPH radical solved in methanol was mixed with 0.2 ml of methanolic extract of the plant sample. Tert-butyl-4-hydroxy toluene (BHT) was used as the control and the negative control was prepared by mixing 2 ml of DPPH solution with 1 ml methanol. After the incubation in the dark for 30 min at 37 °C, the decrease in the absorbance of the reaction mixture was monitored at 515 nm due to depletion of DPPH radical. Antioxidant activity was calculated as described previously^69^.

The ferric ion reducing activity of the methanolic extracts was calculated according to Benzie and Strain^70^ method with slight modification. The reduction of a colorless ferric complex (Fe^3+^–TPTZ) to a blue-colored ferrous complex (Fe^2+^–TPTZ) is monitored by measuring the change of absorbance at 593 nm. A mixture of 300 mM acetate buffer (pH 3.6), 10 mM TPTZ/HCL solution, and 20 mM ferric chloride (FeCl_3_∙6H_2_O) (10:1:1) was used as fresh FRAP reagent. The reaction mixture was incubated for half an hour at 37 °C. The absorption was then recorded at 593 nm. The standard curve was constructed using ferrous sulfate solution (FeSO_4_∙7H_2_O) ranging in concentration from 0.25 to 8 mmol/dm^3^ (0.5–10 mg/ml). Ferric reducing antioxidant power was expressed as μmol Fe^+2^ per g dry weight (DW) of plant material using the calibration curve of Fe^2+^.

### Statistical analysis

The one-way analysis of variance (ANOVA) by SAS software (Cary, NC, USA) ver. 9.3 was used to illustrate the variability among the plant AEPs for each trait. The method used to distinguish among means was Duncan’s test^71^. The results were presented as mean ± standard deviation (SD). Multiple regression analysis was applied using “Stepwise” method of linear regression analysis option of SPSS software (version 25, SPSS Inc., Chicago, Illinois, USA) to determine biological activity and proximate composition associated with morphological traits. Bi-plot graphs of the first two principal components and correlation plots were drawn by Origin 2021 software.

## Data Availability

All data generated or analyzed during this study are included in this published article.
